# The link between cognitive health and neighbourhood: perceptions of the public, and of policy-makers, about problems and solutions

**DOI:** 10.1186/s12889-023-16592-w

**Published:** 2023-09-01

**Authors:** Madeleine Stevens, Tihana Matosevic, Marta Suarez-Pinilla, Sarah Pais, Martin Rossor, Martin Knapp

**Affiliations:** 1 CPEC, Care Policy and Evaluation Centre, London School of Economics and Political Science, Houghton Street, London, WC2A 2AE UK; 2 Dementia Research Centre, UCL, University College London, National Hospital for Neurology and Neurosurgery, Box 16, Queen Square, London, WC1N 3BG UK

**Keywords:** Cognitive health, Cognition, Brain functioning, Neighbourhood, Pollution, Social interaction, Local policy, Public health, Green spaces, Local consultation, Active transport, Public transport

## Abstract

**Background:**

Growing evidence indicates associations between neighbourhood-related factors such as pollution, social isolation and physical inactivity, and cognition, that is, our ability to think clearly, learn and remember. The evidence raises the possibility of neighbourhood intervention playing a role in protecting population cognitive health. However, there is little understanding of these associations among the public and policy-makers, what they mean and how they might be acted on. In this study we explored perceptions of the public and policy-makers about influences of neighbourhood factors on brain functioning, and how they should inform policy.

**Methods:**

Qualitative methods were used in three phases; the study ran in parallel with a quantitative study looking at neighbourhood influences on cognition. In phase one, focus groups were conducted with middle-aged (40–69) members of the public to inform statistical modelling. In phase two, similar focus groups were held in four case study areas chosen based on the modelling results. In phase three, interviews with people in public health and policy roles were conducted, including people in the case study sites.

**Results:**

Participants described effects on their cognition from community, culture and social interactions, access to green spaces and nature, upkeep and safety of the area, and pollution, traffic and noise. Solutions included better local consultation and involvement in policy and planning, support for community interactions and active and public transport, and education on cognition. There was little awareness, but much interest, from local policy-makers and implementers, about links between cognition and place. Barriers to implementation included lack of: effective engagement with local communities, local funding and joined-up health and neighbourhood policy.

**Conclusions:**

People can perceive impacts of neighbourhoods on brain functioning and suggest ways local areas can be improved to support cognitive health. There is support for the idea of population-level interventions to support cognitive health.

**Supplementary Information:**

The online version contains supplementary material available at 10.1186/s12889-023-16592-w.

## Introduction and literature

The relationship between cognitive health and the places where people live and work is of growing recent research interest. Historically the development of public policy has rarely reflected this relationship. Cognition is routinely considered by policy-makers in relation to dementia, particularly in the context of the significant rise in the number of people living with dementia worldwide [1]. However, it is less likely that the link between cognition and place is formally acknowledged within policy. In light of recent evidence that up to 35% of all-cause late-onset dementia worldwide may be preventable by addressing modifiable risk factors [2], there is a pressing need for current policy to address all potential risk factors, including neighbourhood factors. In this qualitative study we sought to understand perceptions, understandings and experiences of the public and policy-makers about relationships between cognition and neighbourhood factors, and to discuss possible ways of modifying the local environment and behaviours to address risks.

Cognition refers to our ability to think clearly, learn and remember [3]. ‘Thinking clearly’ can include planning, organising, concentrating, problem solving, creativity and judgement. While much of the research on prevention of cognitive impairment has been about older populations, cognitive health and brain functioning are essential to health and wellbeing at all ages. Research has shown the relationship between cognitive health and child development [4], mental illness [5] and workforce performance [6]. Cognitive health is a recognised major determinant of quality of life and independence across the lifespan, and impaired cognition carries considerable economic consequences for individuals and society [7].

Quantitative studies have shown links between cognitive health and a number of neighbourhood-related factors including pollution, social interactions and access to nature and green spaces. Among the modifiable risk factors for dementia identified by Mukadam and colleagues’ review [2], some are more obviously related to community and neighbourhood, such as social isolation and physical inactivity, and evidence also links neighbourhood environments to population levels of depression, obesity, hypertension and diabetes [8].

Pollution is one of the key environmental factors that have been linked to cognitive decline. There is evidence of an association between ambient air pollution and the acceleration of dementia-causing processes in older people [9–11] and with brain development in children [12–14]. For those in middle age, there is weaker cross-sectional evidence of a link between air pollutants and performance on neurocognitive tests [15] and cognitive decline [16]. There is also longitudinal evidence of an association between cognition and ambient air pollution [17, 18].

There is less evidence on other neighbourhood stressors and their links to cognition, but some evidence suggests stronger links between air pollution and poorer cognitive performance in the older population from ‘high stress neighbourhoods’ characterised by empty and deteriorating buildings and rubbish [19]. A recent update [20] of a previous systematic review and meta-analysis [21] confirmed, through 35 studies, that multiple aspects of social relationships are associated with cognitive decline, though they also found evidence of publication bias, potentially pointing to an over-estimate of statistical effects. Clarke and colleagues identify greater social and physical engagement in the community as a mechanism through which a combination of individual characteristics and neighbourhood factors such as easy access to public transport, well-maintained public spaces, and community resources are linked to slower cognitive decline [22].

Studies suggesting beneficial effects on wellbeing of access to nature or to green and blue (water) spaces, or even simply exposure to the colour green, have received some publicity. Although studies are of variable quality, reviews suggest there are likely to be health benefits of green spaces on cognitive functioning [23]. There has also been interest in looking at the relative benefits of urban and rural residence [24].

Despite the amount of research emerging around this topic, it remains difficult to rule out other explanations for the associations found, since positive aspects of neighbourhoods are likely to be associated with many other potential explanatory factors, and it is difficult to rule out all confounding factors and reverse causation. If the promise of this growing body of evidence is fulfilled, there is potential for the cognitive health of populations to be protected through improvements to the environments in which people live. However, there seems to be little understanding amongst the public and policy-makers of these connections, what they might mean, and how they might be acted on. In this study we sought to understand whether and how people can perceive day-to-day impacts on their cognition. We explored the perceptions of the public and of policy-makers about local protective and risky aspects of neighbourhoods and about what factors people identify as affecting their cognition. We also explored people’s suggested solutions for creating neighbourhoods more likely to protect cognitive health.

## Methods

This qualitative study set out to address the following research questions:

- What are people’s perceptions of cognitive wellbeing and the effects of neighbourhood factors on their cognition?
- What are thought to be appropriate current or future policy responses to suggested links between environmental/neighbourhood factors and cognition?

The qualitative work took place in parallel with a cross-sectional, statistical analysis looking at relationships between cognition and place in two UK city areas, using UK Biobank data (https://www.ukbiobank.ac.uk/) and reported elsewhere [25, 26]. The qualitative study was initially designed to inform, complement, and aid interpretation of the statistical modelling study running concurrently by exploring people’s experiences and understandings of potentially causal mechanisms.

### Sample and data collection

Data collection and analysis for the qualitative study was in three main phases:

1. Focus groups with members of the public to inform the statistical modelling and the topic guides for the phase two focus groups
2. Focus groups in four case study areas chosen on the basis of the modelling
3. Interviews with people in public health and policy roles, including representatives of the four case study sites.

Recruitment for both sets of focus groups was carried out by a recruitment agency. An age range of 40 to 69 was chosen to match the age profile in the Biobank data, and to target people in middle age who may be aware of cognitive changes and interested in preventing cognitive decline. There were no geographic inclusion criteria for the first set of focus groups. For the second set of focus groups, participants were recruited who lived in each of the four case study areas. Recruitment aimed to include a mix of gender, ethnicity and employment profiles. For the case study site groups, these were matched to the sociodemographic characteristics of the local area as far as possible (See participant characteristics, Additional file 1: Appendix I). Because of the Covid-19 pandemic, focus groups were conducted online. We found that by keeping the groups to 6 participants each, and 2 facilitators, and working flexibly from a carefully prepared topic guide, we were able to develop rapport within the online groups and promote valuable discussion.

First, we conducted three focus groups with members of the general public to explore perceptions of cognition and neighbourhood. We used existing literature and consultation with a broader group of colleagues to develop a topic guide which explored what cognition meant to participants, whether and how people recognised short- and longer-term cognitive changes, what, if any, factors in their environments and day-to-day behaviours they experienced as affecting their cognition, and any strategies they engage in or would consider, to protect their cognitive health (Additional file 1: Appendix II).

These findings, along with those from existing literature, were used to inform statistical modelling, by suggesting factors which could be considered for investigation and to inform development of topic guides for the following phases. A number of those factors for which data were available were investigated in the modelling study and a subset of these emerged as important [25]. The quantitative analyses looked at the relationships between a general measure of cognition and neighbourhood-related and socio-demographic factors. The following covariates were included in models: pollution levels (PM2.5 abs) at home address, percent of green spaces in the neighbourhood, frequency of social visits, self-reported health and self-reported happiness, index of multiple deprivation at home address, sex, age, ethnicity, household income, academic and professional qualifications and job. Pollution emerged as the most important neighbourhood factor related to cognition. Consequently, our four case study sites were chosen to represent different profiles in relation to the relationship between pollution and cognition as summarised in Table 1. We chose to look at four sites where the relationship between cognition and pollution differed, so that we could explore perceptions of the different mechanisms which might lead to differential impacts of pollution, potentially giving insights into protective and harmful factors. Two neighbourhood sites were chosen in each of two major English cities, giving four case study sites in total. Pseudonyms are used for the locations to protect the anonymity of interviewees.

**Table 1:** Profile of case study sites, according to the statistical modelling

Level of pollution	Relationship between pollution and cognition	
	**Worse cognition than expected for the level of pollution**	**Better cognition than expected for the level of pollution**
**Higher pollution**	Innerville	Richby
**Lower pollution**	Edgetown	Leafyton

The character of these four localities is summarised as follows:

- Sites in City A:

Richby is a leafy, affluent area, outside of, but with good links to the City centre. A large majority of the population are of white ethnicity and there is higher than average for the borough proportion of childless households.

Edgetown is a large suburban district further out of the City with relatively high levels of deprivation. A large majority of the population are of black, Asian or other non-white-British ethnicities.

- Sites in City B:

Leafyton is a large, suburban town just outside of the City with high household incomes and low unemployment. It is the least ethnically diverse part of the City.

Innerville is an inner-city, relatively deprived part of the City. It is ethnically diverse with a relatively young population, and higher than average rates of people claiming unemployment benefits.

For the second set of focus groups, two were conducted in each of the four case study sites. The topic guide again covered understandings of cognition, before going on to discuss perceptions of impacts of the local neighbourhood and other factors on cognitive health (Additional file 1: Appendix III). Participants were then briefly told about:

- the factors for which there is evidence linking them to cognition, based on both the modelling study and the existing literature, and
- the headline finding about their area from the modelling study, in particular the pollution finding summarised in Table 1

Participants were asked for their thoughts on the findings, and relevance to the local area was discussed. Finally, participants’ ideas around solutions and barriers to addressing determinants locally were discussed.

Lastly, we conducted 17 one-to-one online interviews with people who worked in policy development or implementation relating to neighbourhoods and/or public health, including people with specific links to policy-making in the four case study areas. Recruitment of people in these roles during a pandemic was challenging, and we tried a variety of methods of approach, including using existing networks to suggest suitable interviewees, and looking on local authority websites to find people in appropriate roles to approach. The professional roles of policymakers taking part are shown in Table 2.

**Table 2:** Policymaker interviewees' professional roles

Code	Position
IN1	Public Health Consultant
IN2	National Health Service (NHS) manager for NHS England
IN3	Knowledge and Information Officer in public health
IN4	Local councillor, cabinet member for tackling inequalities
IN5	Senior Public Health Strategist for health improvement
IN6	GP and academic
IN7	Public Health Strategist
IN8	Public Health Consultant
IN9	Assistant Director for environmental projects, local authority
IN10	Councillor in Edgetown
IN11	Director of Public Health
IN12	Public Health Consultant
IN13	Assistant Public health strategist
IN14	Public Health Consultant and academic
IN15	Director of Public Health
IN16	Director of Public Health
IN17	Head of Public Health Delivery

All focus groups were carried out by MS and TM together, while interviews were conducted by one or other of the two.

### Analysis

Focus groups and interviews were recorded with participants’ prior permission and transcribed by a professional transcriber. Nvivo 12 software was used to manage the data. Data in each of the three phases of the study were analysed separately. Analysis of the first set of focus groups sought factors for potential exploration in the statistical data, and to inform topics to be discussed in, and wording for, the next phase of focus groups.

For both phase 2 (case study site focus groups) and phase 3 (interviews with people in public health and local policy roles), a structured thematic analysis approach was taken, following the steps set out by Braun and Clark [27]. The aim was to produce themes relating to perceptions of factors affecting cognitive wellbeing and how policy can address perceived or possible links between neighbourhood factors and cognitive change. The coding scheme initially consisted of categories drawn from the topic guides and our research questions. Inductive coding was also carried out using techniques from the constant comparative method allowing the development of unanticipated themes; new codes were added as needed. Each category was compared following an iterative process to look for patterns, differences and similarities, further refining existing categories and creating new themes. The software facilitates retention of the link between context and excerpt by allowing codes to be compared across different transcripts, while retaining reference to the sources of data, for example, which enables the researcher to keep in mind the different respondent groups and sites represented.

## Results

Following discussion of what is meant and understood by cognitive health, participants in all eleven focus groups (referred to in headings below as the ‘public’) were able to consider and identify many factors which they experience as affecting how well their brains are functioning. Similar discussions around understandings of cognition took place at the beginning of the interviews with people involved in public health and neighbourhood policy (referred to below as ‘policymakers’). This was followed by discussion of policy barriers and solutions. These initial discussions around the meaning of ‘cognitive health’ were needed to distinguish cognitive from mental health [3].

The remainder of the results section is set out as follows: First, the key themes resulting from the analysis of the eight focus groups with members of the public in the four case study sites are presented in two sections, firstly regarding their perceptions of factors affecting cognition, and secondly regarding their suggested solutions and perceived barriers to implementing those solutions. Illustrative quotes in the ‘public’ section are labelled by the pseudonym of the case study site. The themes are first summarised in Table 3. Second, the key themes from the analysis of perceptions and views of people in local policy and public health roles (based on the 17 interviews) are presented in three sections: cognition in current and future policy; policy responses to the suggested links between cognition and neighbourhood factors; and barriers and facilitators to implementing solutions. Illustrative quotes in the ‘policymakers’ section are labelled by the interviewee code; Table 2 above shows the job role of each interviewee. The policymaker themes are summarised in Table 4.

**Table 3:** Key themes from analysis of the public case study site focus groups

Public perceptions of factors affecting cognition	Community, culture and social interactions
	Access to green spaces and nature
	Upkeep and safety of local area
	Pollution, traffic and noise
Public suggested solutions and perceived barriers	Better consultation with, and participation of, local populations in policy and planning
	Support for community activities and interactions
	Environment-friendly towns
	Active and public transport
	Education on cognition

**Table 4:** Key themes from analysis of interviews with policymakers

Policymakers’ awareness of cognition in current and future public health and neighbourhood policy	Little mention of cognition in public health and neighbourhood policy
	Barrier: definition and understanding of cognition
	Belief that there is a place for cognition as an aim of public health and neighbourhood policy
Policymakers suggested policy responses to links between neighbourhood factors and cognition	Improving public spaces, and infrastructure to encourage active transport
	Community and social interaction
	Access to green spaces and nature
Policymakers: Barriers and facilitators to implementing neighbourhood approaches that support cognitive health	Engagement with communities
	Joined up health and neighbourhood policy
	More local funding
	Research Evidence

### Public: perceptions of factors affecting cognition

Participants described and discussed both potentially harmful and protective factors for cognitive health:

#### Community, culture and social interactions

Social interactions were perceived as important for cognitive health in all case study sites. Positive social interactions were protective, and many examples were given of positive aspects of community, and activities including volunteering were highlighted. It was noted that some people could feel excluded, and that it was more difficult for some people to feel part of the community than others. Participants contrasted their local populations with those in other areas and perceived differences in how friendly people in different localities were. For example, in Richby one group described the local population as more open and having more time to spend on being friendly compared to a different part of the city; Richby was described as having a ‘village’ feel to it.

In Innerville, a far less affluent area, the sense of community and social cohesiveness were felt to create a feeling of security and provide positive psychological benefits. One respondent from a different country felt the area had aided her integration into the UK and she would not want to move elsewhere because of that:

*Having like local places or social places to go to has a massive positive impact … we can take our children and ourselves, we can go for walks, just to kind of refresh our mind and that itself is a positive. I think that has an impact on our mind. [Innerville]*

Local community happenings were felt to be important, including events and entertainment.

A range of factors had helped the cognitive wellbeing of one recent arrival to Richby, especially in the context of the pandemic:

*Next t*o a river and within a community where there’s community events and that has helped me out no end in the last twelve months* … I’m less angry all the time, just the fact that I can walk down, get away from it all, turn everything off and just go and feed some swans for a couple of hours is massive. [Richby]*

The participant saw the change in anger levels as an issue of cognition, something supported by research showing the cognitive underpinnings of anger [28]. The local cultural events mentioned had a more multicultural flavour in Edgetown and Innerville, areas with far higher proportions of residents from ethnic minorities compared to Richby and Leafyton.

The mix of people from different cultural backgrounds in Edgetown and Innerville was described as beneficial and as relating directly to cognition. Cultural, religious and ethnic diversity was experienced as stimulating and people related this to learning and exposure to new ideas:

*I think that when you look at new things and try to grasp and take new ideas, that’s very positive and useful. [Edgetown]*

One participant described the cognitive impact of local culture by describing ‘completely changing’ his feelings and behaviour when travelling from outside into an area with a very strong cultural identity; others agreed.

Elsewhere, a combination of environment-types was described as beneficial for cognition, providing both relaxation and stimulation where there was a mix of quiet areas and sociable areas:

*You can do a 2 minute walk and within that 2 minutes you can be from somewhere nice and peaceful where you kind of like can just reflect and relax and you can kind of clear your mind and then the next thing you can be in the middle of like a buzzy restaurant or café where, you’re kind of thinking a bit more about what shall I do? What shall I have to eat? And it kind of all mixes together. [Richby]*

However, stimulation was not always a pleasant cognitive experience; feeling unsafe was described as heightening awareness, for example when the ‘fight or flight response kicks in’.

While the cultural and social interactions described above were considered beneficial to cognitive health, there was concern that some demographic groups were excluded from those benefits. It was noted, for example, that activities available to the local population in Richby were experienced by some as aimed at a certain better-off demographic, or at those with children. Meanwhile in Innerville, there was concern that young people were not provided with places to go and things to do in the community; it was felt that older generations had more protective community relations. The importance of local provision for promoting physical exercise was seen as beneficial for cognition and conversely closures of activity centres, libraries and youth activities were mentioned in Innerville.

#### Access to green spaces and nature

Parks, rivers, and other green and blue spaces were raised as important for people’s cognition in all the groups. Nature, greenness, space, the opportunity to have some time out, or to interact with others, were all benefits of parks and other spaces which could benefit cognitive wellbeing. This impact was described in various ways including ordering of thoughts, having time out from intrusive or burdensome thoughts, and de-stressing.

*I can sit in the park on a bench, watch the world go by, and the whole thought process changes. You kind of compartmentalise things in your life if you’re stressed or worried about anything, it just seems to make sense sitting in a park, kind of just getting in touch with nature. [Edgetown]*

While breaks outdoors were described as providing opportunities to think, positive cognitive impacts were also conversely described as resulting from focussing on something, exercise, or an activity.

The benefits for many had been highlighted by parks becoming one of the few accessible places during the pandemic lockdowns:

*Colour, as well, can help your mind, it can refresh it, it can make you go on a little journey if you’re sat in a park… even seeing the autumnal leaves now and just seeing the seasons … You don’t realise how beneficial it is for you and for others around you. [Innerville]*

However, the advantages of green and blue spaces were contingent on other factors, in particular litter, upkeep and safety and in the less well-heeled neighbourhoods of Innerville and Edgetown there were more problems raised around use of parks.

#### Upkeep and safety of local area

Rubbish, dirt, maintenance of the local area, and crime were common themes, which people perceived as having negative impacts on their cognitive wellbeing. While rubbish and dirty streets were mentioned in all areas, there was a bigger issue in the less affluent areas of Edgetown and Innerville.

Even in affluent areas parks were affected by rubbish and it was felt that the ‘take your rubbish home’ messaging didn’t work, and more bins were needed, as those available were often overflowing. Some participants blamed residents of local areas for higher levels of rubbish in certain parks, and one focus group in Leafyton blamed residents from Innercity areas for visiting and misusing Leafyton’s parks. However other participants expressed awareness that different areas received different levels of services. One resident of Leafyton had only recently moved from a different area and noted the difference in neighbourhood upkeep and the cognitive impact:

*It’s like they’ve got a team of little elves, and as soon as something gets dumped or there’s something wrong, it’s like they’ll just all scutter out, fix it, and go again, and you don’t even notice that it's been done half the time. But that in itself just frees me more space to think about the stuff that I need to be doing rather than stuff that is an external influence*

It was pointed out that bins locally were easily available and regularly emptied, whereas in the city centre bins were often seen overflowing.

In Edgetown, one of the less affluent areas, a problem with dumping commercial waste next to public bins had led to the local authority’s removal of bins in an effort to reduce rubbish, which had not, it was felt, been successful. Community-organised volunteer litter-picking, often something that had begun during lockdowns, was mentioned in several focus groups.

Fear of crime was raised in relation to both mental and cognitive health. Participants in Richby, which has a riverside area, agreed they felt safe walking around “So… you've got time to reflect” and that they had a’safety net’. The perception of safety, and consequent peace of mine was cited by several as a reason they would stay in the area:

*I would never move away from an area where I can walk alone, if it’s ocean, river, whatever, a lake, something you can walk…that’s very important.*

Whereas perception of neighbourhood decline, antisocial behaviour, littering and fly-tipping were described as stress-inducing and affecting one’s mental health.

####  Pollution, traffic and noise

The above factors were all raised spontaneously as features of the local area having an impact on cognitive health. Pollution was also raised unprompted as a factor affecting cognition in some groups. Where pollution was not raised by participants facilitators introduced the topic, commenting on the research suggesting a link between pollution and cognition, so that all groups discussed the topic of pollution at some point.

The quality of green spaces is affected by pollution; in Innerville, polluted air in local green areas encouraged people to get in their cars, thus adding further to pollution;

*There isn't a lot of open space, there's a lot of traffic, there's a lot of cars, and yeah, that doesn't help your mental health and I can see people getting depressed and that leading to anxiety and that leading to dementia and all that kind of stuff, and be linked into the lack of open space in the area. Even trees for example, there's not a lot of greenery, it's all concrete everywhere, concrete buildings, very little in terms of trees, or nature or greenery. So, that does have an impact. I wouldn't walk in the area, I generally tend to just jump in my car and go somewhere else instead of in the area. [Innerville]*

The relationship perceived between pollution and cognition was not always a direct one:

*There’s too many fumes out there, unless you get to the parks and open spaces it’s really quite suffocating, and I wouldn’t have thought about that as being a cognitive problem but I have anxiety and asthma and that leads to anxiety and that can affect my cognition. They’re all interrelated. [Edgetown]*

It is not just pollution but other aspects of traffic congestion that are perceived as affecting brain functioning, including frustration and difficulty in organising one’s time. In one of the Innerville groups, ‘traffic’ was the first item raised in answer to the question about what local factors might be affecting “the way your brain is functioning”.

People’s experiences of the pandemic had provided the opportunity to experience the local environment with less pollution, less traffic and less noise:

*Certainly the pollution level it’s, as during Covid it was low…*It just made you actually think better, think clearly, reassess life, all those things, just had a clearer sense of vision.* [Edgetown]*

In Richby some participants complained about traffic noise, including ambulance and police sirens, and air traffic noise. Noise was among a number of factors, including hearing about local street crime, that people felt they became used to, but that could still be doing them harm:

*I always wondered if going forward like in a few years time how our brain will react to this noise in the background because we got used to it, I got used to it and now I sleep. But in a few years perhaps how will my spirit, my state of mind be, you know, yeah, I always wondered. I don’t know if it will impact like long-term. [Richby]*

And people noted different responses when noise and pollution reduced during lockdowns:

*During the Covid times when everything was locked down I was kind of missing that noise of the aircraft and I was like literally almost in depression. I’m like “what the hell? Where’s this world gone? There’s no one talking, there’s nothing happening”*.* [Edgetown]*

*The planes come over the house every minute, I mean you can hear them, and once it stopped you felt like you could go out and because the pollution levels were lower and you could go out and, you know, have a nice walk outside when the sun was shining and you just felt so much better that you didn’t hear those planes going over and it was surreal. [Edgetown]*

### Public: suggested solutions and perceived barriers

Having identified and discussed factors which may be affecting cognition, participants went on to consider possible solutions in their local area. People were able to identify areas where things could be improved, and they discussed who should have responsibility. While individuals could take some responsibility for some actions, as well as businesses and community organisations, there were other actions that needed to be taken by local authorities, or central government.

*I suppose personal actions need to change to help relieve pollution, but I don’t think they’ll change unless somebody of authority stepped in and said, “Maybe if we do look at this”. [Leafyton]*

Asked whether more public understanding of the links between local environment and pollution might change behaviours, an Innerville resident commented:

*I think less so for the people on the ground, as in the members that are here, because we’ll always do what we need to do based on our needs and our family’s needs, and then the community needs, but the council and the planners and the organisations that can make long term decisions, if they would factor these kind of things in, then yes. [Innerville]*

Participants had different views about the likelihood of these issues being addressed, and about their being able to influence local policy themselves.

#### Better consultation with, and participation of, local populations in policy and planning

We asked participants how one could go about asking for changes, and who they would contact.

*I would agree that the area is more, I would say neglected, so to be honest I can also agree that I wouldn’t know where to start, because there’s so much going on and there’s so much that needs changing. [Innerville]*

Some participants felt that complaining to the local authority or getting involved in sharing your views would just be ignored. This was sometimes based on experiences of making a complaint, signing petitions, completing a survey, or responding to a consultation. If nothing was done as a result, or the outcome was not the one desired, then some people felt their views weren’t taken into account. This was as true in Richby as in Innerville.

In the more deprived areas there was a feeling among some that their area was not prioritised for action:

*You’ve made a complaint, but are they bothered to help you,* because of the area you live in?* [Innerville]*

Sometimes this was attributed to the ethnicity of the local populace, and sometimes to the geographical position of the area, for example on the edge of a borough (both Edgetown and Richby). In Richby it was suggested it’s “like we’re being punished for being well-off”.

There had been local controversy over local authorities’ attempts to encourage active transport by making it easier to walk or cycle rather than drive for some journeys. Many people, including in Richby and Edgetown, had become active locally on this issue (on both sides of the argument) taking part in demonstrations, petitions, consultations and meetings.

In Edgetown participants suggested that some particular ethnic groups did not know how to make their voices heard, or how to raise complaints (these four participants were from ethnic minorities themselves):

- *1*^*st*^* Pa﻿rticipant*:* With ethnic minorities, they don’t know the right channels to actually complain about things either.*
- *2*^*nd*^* Participant: Or they’re not forceful enough, they don’t become forceful enough.*
- *3*^*rd*^ Participant: *You’re right, yeah, they’re just quiet, they kept quiet and whatever goes goes*.
- *4*^*th*^* Participant:Yeah, and a lot of the literature about it was only in English*

Others were more optimistic about having influence, placing more agency with individuals and communities. One participant in Innerville felt that if everybody starts making their complaints at the same time, change can be brought about. Others in Innerville reported that they had been able to get issues addressed, in this case regarding antisocial behaviour in a particular location:

*We were able to get in contact with the local councillor and with the local neighbourhood police, and they’ve actually been able to put signs up, and it has made a tremendous difference with doing this. So, yes, but you have to, as an individual, be patient to communicate.*

It was pointed out that where there were more transient populations, it was harder to get community efforts together for action:

*I think the community has changed quite a lot. We’ve got a lot of transient people and the majority of people are renting or not there permanently, so I think they don’t take that kind of care in their local area as local residents do who are permanent residents. [Innerville]*

There was a strong feeling however, that people would be willing to help with things and volunteer, if the local authority communicated more and it felt like a supported and joint project:

*[In the park] they’ve got the play centre. They should use that and involve us, maybe – volunteers; we’ll come and help and do bits and bobs. There’s a lot of people here – women, people, men, as well – will be happy to volunteer and help out to improve, but the council, they won’t. [Innerville]*

While some had very oppositional attitudes towards the local authority, particularly in one of the Richby groups, many participants had ideas for more meaningful consultation:

*Have something where you can express and somebody works as a go-between [Innerville]*

*Maybe set up working parties with your constituencies and make sure that everyone has a voice, if they’re not physically able to then do it online or there just has to be some way that everyone’s engaged*.* [Edgetown]*

*Maybe we can have a few people going around individually for the people that can’t come out… to really get everyone’s views and then you know that everyone’s been catered for*.* [Edgetown]*

#### *Support* for community activities and interactions

Action to support community interactions and address loneliness were felt to be important:

*It might sound simple, but communicating with different people can lift your spirits, talking to ones about different experiences that you’ve been through. You sometimes think you’re the only one going through it, but by communicating and speaking to others, it takes your mind off yourself and then you can be actually helping somebody, as well. [Innerville]*

People had lots of ideas, and existing examples, of actions that could be taken by residents to improve the local area, and the lifestyles of those living there, including clearing-up groups, community gardens, incentives to encourage young people into parks, community vegetable plots, (Innerville) free-to-use venues for the community, spaces for young people, cultural events, choirs, gardening (Richby), more clubs to involve people in the community who may be lonely, the elderly, the young, the unemployed, social spaces to sit in (Edgetown).

*They could have community centres whereby they can educate ones to grow their own fruit and veg…I do think it’s all about educating, too, one’s mind. So they could have little workshops where they can improve and let ones know the benefit of how their input can have a big impact with the environment – nature, birds, and things like that. [Innerville]*

Some very specific ideas were mentioned, including identification of particular vacant plots or buildings which could be used for the community.

Some said that while some of these things existed there needed to be better information sharing and more effort to involve those who may perceive barriers to attending, or not be aware. Ideas for places to better advertise existing or new provision included local newspapers, flyers, community-spirited people extending invitations, the local authority website, local libraries, GP surgeries, local radio, social media and word of mouth. While in Richby someone suggested that there was little in the way of free provision locally, with most classes and activities quite expensive, participants from Edgetown were positive about the amount of local provision and efforts to enable people to attend social activities. More could be done though:

*The Council needs to take into account the elderly people that don’t get out of the house and … have strategies to sort of help them to communicate and engage with other people because otherwise what’s going to happen? They’re going to have all these health problems, especially with dementia, Alzheimer’s and Parkinson’s, and that impacts the NHS. [Edgetown]*

#### Environment-friendly towns

In all sites, the benefits of shopping locally were raised as a desirable solution to some of the negative impacts on cognition. People needed to be able to find the items they wanted locally and this could promote feelings of inclusion, such as availability of halal food. People pointed out that foodstuffs available locally were not always conducive to a healthy diet, and that this was also likely to affect cognitive health.

*In our local high street …we have a lot of takeaways, which are relatively cheap, so it's easy to grab a quick bite, but then when you look at like parks and gyms, we don't have a lot of those… compared to how many takeaways we have. [Innerville]*

There were concerns in Leafyton that the main town centre was going downhill, and was no longer a desirable destination, but it was felt that smaller local areas were developing independent cafes and shops which made them attractive places to go, and enabled travel by foot instead of by car.

The same was true of improving green spaces, which could have multiple benefits, reducing the need for travel, improving the air, and facilitating community interactions:

*If the parks were safer, where women could go, mothers could go, families could go, it'd alleviate the problem of having to get in your car … and that'd ease up on the pollution. And if you got clean park areas, or green areas, you should theoretically have trees there and that'll help to clean up the air as well. So, if they can tackle the streets and the parks that would be a big one for me. Innerville*

There was strong support for protecting green spaces, and some positive comments regarding local authorities doing so, though safety, cleanliness and antisocial behaviour needed to be addressed. Solutions suggested included higher visibility Park rangers or Park Keepers; more than one group commented that these used to exist and should be brought back to maintain the quality and safety of parks.

*I mean when I was growing up you wouldn't cheek one of the park keepers [Laughs] you'd get a clip round the ear hole. [Innerville]*

Other desired solutions included more street cleaning, more bins (including in parks), emptying bins and clearing rubbish quickly so it did not become a magnet for other rubbish, free bus to the park, bringing back the planting and maintenance of flowering plants and free park-based activities for teens.

In Richby, a participant noted an inequality in provision of street trees, and likely impacts on factors affecting cognition:

*They’ve got more trees on their streets than some of the, and again it does come down to this sort of class barrier, that some of the less affluent areas don’t have as many trees as the more affluent areas, you know, of the Borough. And that does help because they absorb the impurities, they also absorb the noise as well, you know, so I think that does have an impact. [Richby]*

#### Active and public transport

In general, there was agreement that many people needed to drive less and that other options should be supported; climate and environmental issues were referenced as well as cognition, and some felt passionately that more and quicker action was needed. Local solutions suggested included improving public transport, schemes for only letting some number plates drive on some days (as happens elsewhere globally), planting more trees, park and ride, more working from home, subsidising electric cars to address affordability and providing charging points. In Leafyton, one participant suggested that families should consider limiting themselves to one car per family. People also pointed out the difficulty of adjusting existing infrastructure. In Edgetown, a single, congested, main road going through the area was unavoidable to reach most destinations:

*The only alternative for people who don’t drive, like me when I gave up my car, is the buses that have to run along that one main road, so some days you actually feel like you’re under siege because you can’t get out of the area. [Edgetown]*

Again, socioeconomic inequalities were referred to. One participant in Leafyton had an insight that it was easier for people in more affluent areas to reduce their reliance on the car because, a) they had more room for a home office so could commute less, and b) the schools were more likely to have smaller catchments and so easier to get to without a car. The participant pointed out, however, that too many people nevertheless brought their children to school by car, and another participant referred to the example of the local grammar school where children came from far and wide.

Financial disincentives were discussed, and the importance of making public transport cheaper than driving, for example through congestion charges and more parking fees and fines, with the money being put back into the area (Innerville). One participant in Edgetown argued a counterpoint, that cheaper public transport might stop people from choosing cycling and walking, and that active transport should be incentivised.

Efforts of local authorities to encourage active transport, which were accelerated in some areas during the pandemic by restricting routes for car drivers, were controversial. And while there was acceptance that making it easier to walk and cycle was a good idea, some objected to additional restrictions over where they could drive, with some suggesting that diversions and blocks could lead to more traffic and more pollution. Others responded positively to developments elsewhere and wanted the same for their part of the city:

*It's happening in quite a few parts of the City where they'll block the end of a road off, just to stop cut-throughs, and then that does encourage people to walk instead of taking the car. [Innerville]*

However others were less optimistic and felt public transport, walking and cycling could feel unsafe whereas people felt safe in their cars:

*They’ve given us cycle lanes, a little bit here and a little bit there…but they’ve not seen it through. I’ve not seen anybody use the cycle lanes – maybe one, maybe two – in about five years, but not local people. [Innerville]*

*Even if it takes them longer, they will still drive because they're in the enclosure and comfort of their car and they feel safe, whereas if you walk, you're then subject to, you know, potential gangs and harassment. [Innerville]*

Others spoke of the need to make public transport more pleasant:

*Sometimes it is slower to get around in your car than it is on public transport and I can make my journey from here to work on public transport in 20 minutes and it used to take me more than an hour by car but on public transport, it is extremely unpleasant. [Edgetown]*

Not everyone had good public transport links, though many did. Improvements wanted included higher frequency, cheaper, safer, more connections and more routes.

#### Education on cognition

We asked participants whether they thought more education of the general public on links between local factors and cognition would affect people’s behaviour. People agreed that awareness and education on the issue was worthwhile.

*The more we get to know about it, the more you will register and you will make those changes*.* [Leafyton]*

When the question was framed in terms of potentially reducing the risk of dementia (a link one participant had already read about) agreement that the evidence might affect people’s behaviour was particularly strong:

*I think obviously dementia affects a lot of us, and families obviously. So, I think if obviously that education was out there it would one hundred percent have an effect on the people I believe. [Leafyton]*

It was suggested however, that people might learn best by feeling the benefits themselves, rather than simply being told about them, for example trying out exercise and social groups, and active modes of transport.

*I think once they try it they’ll actually realise that doing is better than reading [about it].*

Better understanding could help policy-makers and the public build healthier communities:

*We’re just becoming more aware of it…And the brain’s a mighty complicated piece of kit, isn’t it?*

### Policymakers: cognition in current and future public health and neighbourhood policy

Interviewees reported that, as far as they were aware, cognition did not appear in policy documents and discussions related to public health and neighbourhoods. The only exceptions were occasional references to dementia, or to people with learning disabilities, but this was in relation to specific policies or interventions for people living with those disabilities and not to prevention of cognitive decline. ‘Wellbeing’ did appear, and some interviewees suggested that cognition could be understood to be part of wellbeing in these contexts. A number of interviewees noted that existing health promotion messages and prevention frameworks designed to address risk factors for multiple conditions, including heart attacks and vascular disease, could also be relevant for targeting cognitive health.

*A lot of the prevention elements are the same, so you know, healthy weight, healthy diet, being physically active, … so, I think there are preventative programs in place that may well be impacting on cognition, but it's not explicit. (IN16)*

Following discussion of emerging evidence on cognition and place, presented briefly by the interviewer, interviewees agreed that preventing cognitive decline ought to form part of public health policies. Barriers to the incorporation of cognitive health as an aim of public policy were raised by interviewees. The lack of a clear definition, and of popular understanding, were barriers to including cognitive health in everyday conversations about policies on green space, pollution, and community interactions:

*I think there's some work to do in terms of like what the word even means ‘cognition’. It's a very technical term, I know what it means being a professional. But you know, even the idea of mental health is taking a long time to understand. And we still haven't got parity of esteem in terms of mental and physical health. (IN8)*

### Policymakers: policy responses to suggested links between environmental/neighbourhood factors and cognition

#### Improving public spaces, and infrastructure, to encourage active transport

Policy-makers suggested many of the same policy solutions as the public focus groups with active transport (improving walking and cycling infrastructure) prominent. The concept of “15-min communities” was mentioned [IN5] as a way to reduce car dependency; this is the idea of developing communities where the local population can access everything they need within 15 min’ walk from their home (e.g. good quality and value shopping, schools, healthcare). There was also support for continued efforts to develop low traffic neighbourhoods (LTNs), alongside an acknowledgement from an Edgetown respondent that rushing LTNs through during Covid, had not turned out well, due to the lack of time for planning and consultation:

*I think the thing that annoyed most people was that they weren’t asked, it was imposed… But we’ve now made it a point that we consult on everything* (IN4,* Edgetown’s borough)*

Respondents pointed to other existing policies such as clean air zones and campaigns to encourage people to be more active by walking and cycling. It was pointed out [IN10] that, across the borough of which Edgetown was part, a high percentage of car journeys were under a mile, meaning there was much scope for changing habits. Policy interviewees concurred with public participants that a lack of adequate infrastructure was a barrier to active transport, including the standard of pavements, which could pose a risk for pedestrians; one interviewee noted that cuts to service budgets had been addressed by widening the size paving cracks could reach before intervention was required.

One respondent was implementing policies aiming to make street spaces more attractive and thereby more used:

*We want to make public space actually public space and not just a travel route from A to B. We’re much more about what the journey is. Is it good to, is it great to have a walk on a well-lit vibrant street, would it encourage you to do that more often? Well I think the answer is yes it will. If streetscape’s right people will come and they will use and they will want to use again and again. (IN9)*

#### Community and social interaction

Many interviewees saw community interactions as important for wellbeing, and some identified that this was already a focus of policy. The interviewee quoted just above went on to explain their desire to think about and use spaces in a different way to bring people together [IN9] through the built environment and by encouraging social events. One policy interviewee in Edgetown recognised that the local authority needed to be better at communicating with their local communities when it comes to social events. Speakers of the dominant local minority ethnic language were comparatively well catered for through a local radio station. However:

*Everybody else is sort of a little bit peripheral to that, so they’ve tried to open interfaith communities, but if you’re not from a faith community then you miss out on that as well, so it’s a tricky one to kind of navigate really…we’ve been struggling a bit with that too. (IN4, Edgetown)*

#### Access to green spaces and nature

There were high levels of awareness of the importance of nature and green spaces for people’s wellbeing and people did not have difficulty imagining that these might also have an impact on cognitive health. Some could point to existing policies promoting access to nature. Respondents were aware of the importance of nature and green spaces for the wellbeing of their populations. Spaces were reported as well used for individual enjoyment as well as for social interactions, particularly during and following the pandemic (e.g. IN4). Specific policy initiatives were named such as ‘green prescribing’ (IN14), a branch of social prescribing, encouraging people to get into nature to improve their mental health. During the pandemic, initiatives were taken online but maintained a link to nature – such as a sunflower growing group for people in sheltered accommodation (IN8).

One respondent was interested in the public perception (reported from the focus groups) that park rangers were no longer present. He explained that the role had changed and expanded, but also referred to the budget implications:

*In the old days the Park Ranger will be sat on a park bench and you'd really see them. The Park Rangers now do nature education classes for schools and they do outreach to schools to talk about green space and nature…But there are less of them because* … *The reality is you can have that, or you can have care assistants. (IN11, Innerville)*

### Policymakers: barriers and facilitators to implementing neighbourhood approaches that support cognitive health

#### Engagement with communities

We saw that our public focus group identified better quality involvement of the community and more meaningful consultation about neighbourhood changes as important for bringing about effective and acceptable change in communities. Our policymaker interviewees concurred, recognising that Councils need to be better at listening and engaging with local communities.

*Councils [need to] think about sort of a less top down approach to running things, and sort of using, trusting communities more to be motors of change, and as like, you know, people who have real ideas of where they live, and legitimate desires, right, so like when people say I gave my opinion but it wasn’t listened to, it’s frustrating, and I completely empathise with that frustration … (IN10)*

An interviewee in Edgetown felt that participants could be paid for engagement in community development and consultation projects, and that there could be investment in developing the skills of local people to get involved in planning (IN10). It was felt that there was still an uphill battle to get more people engaged in community discussions:

*Meaningful community engagement is one of the, or the hardest things that local governments can do… I think very few people understand the kind of the powers of response, and responsibilities of local government and …kind of expect local Councils to do something when we can’t, or don’t understand the kind of day-to-day successes that local Councils might go through. And I don’t know how I, yeah, I wish I knew how to solve this (IN3).*

However, in terms of public health approaches, at least one correspondent had some optimism, speaking of the opportunities that had been afforded by the Covid pandemic, for bringing public health messaging into marginalised communities:

*To understand sort of some of the barriers, so we’ve done a lot of engagement with different sort of community groups, … we’ve gone into those groups and spoken to them and, you know, taken information … so we’re much better I think now.(IN5)*

One correspondent from a non-case study inner-city borough reported strong and consistent efforts in getting local populations involved in designing enjoyable community spaces and neighbourhoods:

*We’ve moved a long way from just turning up and doing stuff and then leaving, now it is really much a discussion and trying to find out what is the best route to change people’s environment around them.(IN9)*

As well as consulting, communities were kept informed:

*We talk about what we’re doing, show the photographs of why we’re doing it, explain why we’ve done it the way we do and then there’s this open conversation, you know, if we’ve got a problem we tell them but we explain why we’re doing things and why we’re doing that to their particular bit of road and what the benefits for them are*

Better community engagement may result, it was suggested, with a greater emphasis in policies on collective responsibilities as opposed to current focus on individual actions:

*I think it’s important to find a really good balance between narratives of personal responsibility and collective action. I think a lot of public health efforts are still very focussed on personal responsibility…how can you do things better for your mental health, how can you lose weight, how can you quit smoking, and a lot less of it is like oh, how can you empower your community to protect your local cycle lane. (IN3)*

#### Joined-up [health and neighbourhood] policy and reducing bureaucracy

Solutions based on closer working between local government departments were mentioned but a lack of joined-up strategic policy planning and implementation was seen as a barrier to neighbourhood preventative efforts. Such efforts might involve, for example, the built environment, traffic and health promotion and broader preventative policies that might include cognition. Without joined-up approaches and thinking it was difficult to take some ideas forward, such as ‘spaces for all ages’, aimed at providing accessible and appropriate environments for all:

*… if like we try to talk to, you know, housing, there was suddenly like a ‘what do you mean designing for older people and toddlers in mind?’*

There were also barriers between local government and the NHS:

*The way we work with the NHS, there’s a lot of administrative argy-bargy … that’s something that I think is a major barrier that you’re having to go through all these different layers of bureaucracy to get to the actual doing bit from the ground, so yeah. I would say that’s a major barrier as well as money.* (IN4).

There was a suggestion that including cognition might contribute to improving collaboration between policy areas.

*I think there’s a general need to, especially in public health, to move away from clinical and into kind of more holistic thinking and to integrate between different departments or different types of policies, I don’t know, I’m thinking like housing, education, green space, built environment. I think my take on it is whatever words are powerful enough to do that are useful, so if cognitive health is a term and an idea that people can rally behind then it’s useful. (IN3)*

One inner-city public health strategist felt there needed to be more emphasis on health in all policies as part of a more holistic approach to community planning. He added:

*There's a lot of discussion about the ‘whole systems approach’ or ‘health in all policies’, so I think that's pretty good…You have to keep in mind that, if you are building a new neighbourhood, you should have access to a park…. There should be kind of a distance from main road so that you try and avoid some sort of pollution to the people who are living there. Try and build some plants and trees there which would grow maybe 10-15 years down the line, and they would provide that barrier in terms of pollution and be giving the healthy lifestyle in general. (IN13)*

#### Funding

Insufficient funding was highlighted as a key barrier to implementing changes that local governments would otherwise be keen and able to make, by six interviewees:

*[Local government] has huge capability and the ability to also shape place and lots of good policy ideas. The biggest problem that we have is austerity over the last ten years (IN17)*

*60% of our funding has been cut from central government, we are literally pumping whatever we get into supporting our most vulnerable, it all goes on social care, it all goes on supporting our disabled, supporting our kids, and then on top of that we have a refugee issue where we had a large influx of refugees who’ve come in, so they are obviously getting our first priority, at the expense of things that, we’d all like to do, but we’re just not able to. So our rubbish bins are collected every other week rather than every week, we would like to do it every week but it’s just money, so give us the money and we’re more than happy to do it. (IN4)*

#### Research evidence

There was some awareness of a link between pollution and cognition prior to interviews, and much interest from policy-makers:

*That’s so interesting …I never knew that there were links, environment links and I consider myself relatively well read! (IN10)*

*I haven’t heard of that, so thank you for educating me on that …we’ve got somebody in our team who looks at air pollution and we’ve got someone else who’s working in dementia, so yeah, it’s definitely something that I can bring back to the team to say “did you know that air pollution increases the risk of dementia?” (IN5)*

One interviewee had heard of a link between pollution and dementia (IN16) but did not think cognition was mentioned in existing air pollution strategies, including policies to reduce pollution around schools. Another had been hearing about the link with dementia “more and more, and it’s personally terrifying” (IN3). As with the public, there was a view that people may take risks to their cognitive health seriously:

*I think people are more inclined to take better health if it’s linked to their brains rather than physical health in some ways (IN5)*

Policymakers spoke of public confusion over public health messaging however, and the need for reliable evidence and clear messaging that could combat ‘fake’ information (IN13).

Our interviewees were interested in the research and felt that trustworthy evidence on the links between neighbourhood factors and cognitive health would be valuable for informing policy and bringing the public along with neighbourhood enhancements.

Evidence supporting the potential for cost-savings could be of use:

*You could stretch that to our industrial strategy, making a workforce that’s fit for the future, and then if you get to the older end, you could say, “I’m preventing hospital admissions, I’m saving care costs”, but for people themselves, I guess we’d have to hang it on, yeah, I guess you’ll have more years in good health, kind of thing. No one wants to get dementia, isn’t it?* *(IN15)*

Our findings suggest relatively little use of academic research in local policy discussion:

*Maybe if we were really starting something new there would be but I suppose so much of what we do is just ongoing, you know, or it’s stuff that seems like a no-brainer so people don’t dig into the research because it just seems obvious that, you know, we should be improving access to X, Y, Z, we should be reducing people living in damp overcrowded households, like do, you know, maybe that doesn’t feel like there’s a need to thoroughly review the evidence. (IN7)*

However, evidence of environmental risk factors for dementia would be of interest to policymakers, interviewees said, and could also potentially impact people’s behaviours:

*Dementia for sure is a real issue in our communities, so I think if that link was made as well I mean I think it would definitely be important for policymakers to take that in mind, that’s essential. There’s already a movement towards, you know, trying to make …the air less polluted, so it would add greater weight, but things are going in that direction anyway because there are clear links, scientific links between general health and public health, and air pollution*. (*IN10*,* Edgetown)*

While some interviewees thought that evidence on the harms of pollution were already well received and acted upon by policymakers, others felt that adding cognition to making a case for reducing air pollution could be beneficial.

While cognition had not been much thought about thus far in relation to place, interviewees saw potential for this to change, and that research could contribute to this impetus.

## Discussion and conclusions

Our study set out to explore people’s perceptions of the impact of their local area on their cognitive wellbeing. The study was motivated by wanting to take another approach to exploring and understanding connections suggested by statistical analyses; we wanted to hear from people living in those areas for which we had some statistical findings to see if they could identify impacts on their own cognition and perhaps illuminate possible mechanisms by which local factors may influence cognition.

The four sites were chosen based on the findings related to cognition and pollution, and not because of their contrasting sociodemographic profiles. Richby and Innerville had high levels of pollution compared to Leafyton and Edgetown, but the effects of pollution on cognition were high compared to areas with similar levels of pollution in Innerville and Edgetown, and comparatively low in Richby and Leafyton. Despite controlling for factors including age and income, the sites were clearly distinguished by demographic characteristics, with Richby and Leafyton’s populations relatively affluent and of majority white ethnicity, in contrast to those of Edgetown and Innerville. Focus group members were varied socio-demographically, chosen broadly in proportion to local demographics. It is evident that sociodemographic factors, beyond what the modellers were able to control for, are associated with the differential results in these four areas. The qualitative work was able to suggest possible explanations for these impacts. In affluent Richby and Leafyton, better upkeep of the area and lower fear of crime appeared to be among possible explanations for lower effects of pollution on cognition, compared to Innerville and Edgetown. The quality of green and blue spaces available within walking distance also may have been better. Many participants referred to socioeconomic inequalities, and some participants felt their areas were relatively neglected by their local authority. There is a great deal of research linking aspects of deprivation to physical and mental health outcomes [29, 30]. The discussions reported above raised many of these issues and linked them to cognitive health. The strong appearance of themes around rubbish and area maintenance recalls ‘broken windows theory’ which posits that swift repairs are needed to prevent decline (for a recent review of evidence see [31]).

The UK government has recently published results from a review of nearly 70 quantitative studies by the Committee on the Medical Effects of Air Pollutants, concluding that it is likely that air pollution contributes to a decline in mental ability and to dementia in older people [32]. Recent research has also shown effects of poor air quality on short term declines in cognitive functioning [33]. In our study, while not all participants immediately considered pollution as one of the main environmental factors affecting their cognition, others did, although the relationship was not necessarily a direct one. All accepted the evidence as likely to be correct, with policy officials suggesting that stronger evidence could help build the case for more investment in changing the built environment and transport strategies to aim for healthier communities. Pollution was perceived as a bigger problem in Innerville than in Richby, partly perhaps because it was harder for residents to travel to less polluted areas.

The interview and focus group formats, as compared to a survey for example, allowed the necessary clarification of people’s understanding of cognition, both at the beginning and during discussion of related factors. The findings went beyond exploration of the statistical results. Participants were interested in engaging with and discussing their cognitive health. They could describe temporary changes in their cognition and could relate aspects of their neighbourhoods to cognitive change in themselves, including feelings of stress and wellbeing. People were keen to suggest and discuss improvements to their local areas which could support cognitive health. The interviews and focus groups took place during the second year of the Coronovirus pandemic and people had recent experience of an abrupt alteration to their lifestyles. This provided a natural experiment of sorts, where individuals could be their own case controls, reflecting on enforced changes to their lifestyles.

We had originally planned to conduct the case study focus groups in person, in the neighbourhood, but were not able to do so due to the pandemic restrictions.; the common experience of being in and travelling to a community venue could have provided interesting angles to explore. While this could be considered a study limitation, and the online format perhaps resulted in more ‘turn-taking’ in comparison to in-person groups, people participated well in the online groups.

People identified protective aspects of their neighbourhoods, as well as risks. Participants in our Edgetown and Innerville focus groups were positive about ethnically mixed communities such as their own, and felt that community integration was important, and could help prevent isolation and aid cognitive wellbeing. Ethnic density research has suggested there are beneficial effects on mental health problems of being in a community where others of your ethnicity are also represented, though the mechanisms are not well understood and cognitive wellbeing has not yet been considered [34]. In our groups, the mix of ethnicities was presented as beneficial and stimulating.

A strong theme in our study was that of meaningful consultation with local communities. Low Traffic Neighbourhoods were rapidly introduced during the pandemic in some areas because of fears that people turning against public transport would lead to an overwhelming increase in car use and pollution. These schemes were divisive and controversial locally and many schemes were reversed. A key learning point for local authorities was that high quality public consultation was needed [35]. A growing body of research into democratic innovation explores methods for how citizens can be more meaningfully involved in policy-making (see for example [36, 37]).

The suggested solutions emerging from public and policy participants in the current study are already the subject of a great deal of research relating to healthy neighbourhoods, addressing physical and mental health outcomes including obesity and loneliness. The idea of improving population wellbeing by improving urban environments is not new, though societies may seem to have made little progress in implementing approaches suggested by Duhl and others since the 1960s [38]. Restorative Cities, Spaces for All Ages and Socially Inclusive Architecture are among more recent conceptions and approaches aiming to create environments conducive to better physical and mental health, and potentially to preventing need for personal social services [39, 40]. Cognition is rarely highlighted in the rationale for such interventions. However, parallel developments are the emergence of ideas and guidance for dementia-friendly communities [41] and dementia-inclusive societies [42]. Incorporating ideas about prevention of cognitive decline, through addressing factors which can protect brain health, could contribute to a broader approach to improving population wellbeing. Continued progress in usefully implementing the findings of research requires an understanding of political forces, the disparate and diverse roles, actors and interests that influence decision-making; scholarship linking political theory and public health can support understanding the complex networks that influence, and could influence, public health policymaking [43].

Evidence on the economic benefits of preventing cognitive decline can only add to motivation to address these problems. The economic case for investing in the prevention of physical and mental health conditions in the UK has been presented in various studies (e.g., in relation to mental health [44]). A costs and cost-effectiveness modelling study has suggested large savings would result from implementing some interventions which reduce the prevalence of dementia. The highlighted interventions, for which some evidence of effectiveness exists, are treatments for stopping smoking, provision of hearing aids, and treatment of hyper-tension [2]. Evidence linking neighbourhood characteristics to dementia is growing, as we have seen, and could potentially help demonstrate the economic case for faster moves towards healthier neighbourhoods [45].

Given continued poor public understanding of the possibilities for prevention of dementia, Alzheimer’s Research UK is promoting a new way of thinking and talking about protection of our cognitive health, by talking about Brain Health rather than dementia [46]. They found people were much more likely to believe that they could influence their brain health, than they were to believe they could influence their risk of dementia. Our study, based on conversations with people in middle age, and with policy-makers, showed that people could indeed identify effects of their neighbourhood on their brain functioning, and could suggest ways their neighbourhoods could be improved to support cognitive health. While there are growing efforts aimed at getting individuals to take more note and responsibility for their own brain health [3], an effort supported by many of our participants, our study adds to efforts suggesting environmental and population-level interventions have a key role to play in efforts to support the population’s cognitive health.

### Supplementary Information

**Additional file 1: Appendix I.** Composition of phase one and phase two focus groups. **Appendix II.** Main questions asked in Phase one topic guide. **Appendix III.** Main questions included in Phase two topic guide.

## Data Availability

The data cannot be made available publicly due to an ethical restriction as the consent of participants implied that only the research team will have access to the data provided for the study. Anonymised data from the study is held by Dr Madeleine Stevens. Those interested in obtaining the data and study materials should contact Dr Stevens to request appropriate approval for access.

## Abbreviations

PM2.5 abs: The absorbance of particulate matter < 2.5 μm
GP: General Practitioner
NHS: National Health Service
