# Supplementary material for: The link between cognitive health and neighbourhood: perceptions of the public, and of policy-makers, about problems and solutions
Source: BMC Public Health. 2023 Sep 1;23:1694. doi: 10.1186/s12889-023-16592-w (PMC10474713; doi:10.1186/s12889-023-16592-w)
Supplement: Supplementary file 1 — Additional file 1: Appendix I. Composition of phase one and phase two focus groups. Appendix II. Main questions asked in Phase one topic guide. Appendix III. Main questions included in Phase two topic guide. [file 12889_2023_16592_MOESM1_ESM.docx]

**Appendices**

**Appendix I: Composition of phase one and phase two focus groups**

Phase one focus groups, participant characteristics

| **Participants** | **Age group** | **Gender** | **Ethnicity** | **Highest qualification** | **Social grade** | **Live** | **Lived at current address** |
| --- | --- | --- | --- | --- | --- | --- | --- |
| Focus Group 1 | 50-59 | Female | White British | GCSEs or equivalent | C1 | Rural | 20 years |
|  | 40-49 | Male | White European | Degree or equivalent | C1 | Urban | 4 years |
|  | 40-49 | Male | British Asian | Post-graduate degree or equivalent | B | Urban | 15 years |
|  | 40-49 | Female | White British | A-Levels or equivalent | C1 | Rural | 4 years |
|  | 40-49 | Female | White European | Degree or equivalent | C1 | Suburban | 4 years |
|  | 40-49 | Male | White British | A-Levels or equivalent | C1 | Suburban | 6 years |
|  | 50-59 | Female |  |  |  | Urban |  |
| Focus Group 2 | 40-49 | Male | White British | Degree or equivalent | E | Suburban | 35 years |
|  | 60-69 | Female | White British | Degree or equivalent | C2 | Rural | 20 years |
|  | 60-69 | Female | White British | GCSEs or equivalent | C2 | Suburban | 12 years |
|  | 60-69 | Female | Black British | Post-graduate degree or equivalent | C1 | Urban | 18 years |
|  | 50-59 | Male | White British | Degree or equivalent | C1 | Suburban | 21 years |
|  | 40-49 | Male | White British | Post-graduate degree or equivalent | C1 | Urban | 7 years |
| Focus group 3 | 60-69 | Male | White British | A-Levels or equivalent | C1 | Urban | 40 years |
|  | 40-49 | Female | Black British | Degree or equivalent | C2 | Urban | 3 years |
|  | 60-69 | Female | White British | A-Levels or equivalent | C1 | Suburban | 4 years |
|  | 60-69 | Male | White British | GCSEs or equivalent | C2 | Rural | 3 years |
|  | 60-69 | Male | White British | Degree or equivalent | C1 | Suburban | 30 years |
|  | 40-49 | Female | Indian | Degree or equivalent | C1 | Suburban | 8 years |

Phase two (case study site) focus groups, participant characteristics

| **Focus Group** | **Age** | **Gender** | **Ethnicity** | **Highest qualification** | **Lived at current address** |
| --- | --- | --- | --- | --- | --- |
| **Innerville** |  |  |  |  |  |
| Focus group 1 | 40-49 | Male | British Asian | Post-graduate degree or equivalent | 6 years |
|  | 40-49 | Female | British Asian | GCSEs or equivalent | 30 years |
|  | 50-59 | Female | White British | GCSEs or equivalent | 17 years |
|  | 40-49 | Male | British Asian | GCSEs or equivalent | 15 years |
|  | 40-49 | Female | British Asian | Degree or equivalent | 20 years |
|  | 40-49 | Female | British Asian | Degree or equivalent | 15 years |
| **Innerville** |  |  |  |  |  |
| Focus group 2 | 40-49 | Female | British Asian | A-Levels or equivalent | 14 years |
|  | 50-59 | Female | Mixed White/Black Caribbean | A-Levels or equivalent | 30 years |
|  | 40-49 | Male | British Asian | Degree or equivalent | 9 years |
|  | 40-49 | Female | British Asian | GCSEs or equivalent | 40 years |
|  | 50-59 | Female | White British | GCSEs or equivalent | 25 years |
|  | 40-49 | Female | Asian | Post-graduate degree or equivalent | over 40 years |
| **Leafyton** |  |  |  |  |  |
| Focus group 1 | 67 | Male | White British | A-Levels or equivalent | 16 years |
|  | 50-59 | Female | White British | A-Levels or equivalent | 21 years |
|  | 50-59 | Male | White British | GCSEs or equivalent | 32 years |
|  | 50-59 | Female | British Indian | GCSEs or equivalent | 20 years |
|  | 40-49 | Female | White British | GCSEs or equivalent | 1 year |
|  | 40-49 | Male | White British | A-Levels or equivalent | 10 years |
| **Leafyton** |  |  |  |  |  |
| Focus group 2 | 50-59 | Male | White British | A-Levels or equivalent | 25 years |
|  | 40-49 | Male | White British | A-Levels or equivalent | 2 years |
|  | 40-49 | Female | White British | A-Levels or equivalent | 15 years |
|  | 40-49 | Female | White British | A-Levels or equivalent | 7 years |
|  | 50-59 | Female | White British | GCSEs or equivalent | 20 years |
|  | 40-49 | Male | White British | Degree or equivalent | 20 years |
| **Richby** |  |  |  |  |  |
| Focus group 1 | 40-49 | Male | White European | Degree or equivalent | over 2 years |
|  | 40-49 | Female | White European | Degree or equivalent | over 2 years |
|  | 40-49 | Female | White European | Post-graduate degree or equivalent | 3-4 years |
|  | 50-59 | Male | White British | Degree or equivalent | 15 years |
|  | 40-49 | Male | British Mixed Race | A-Levels or equivalent | 12 years |
|  | 40-49 | Female | British Indian | Post-graduate degree or equivalent | over 5 years |
| **Richby** |  |  |  |  |  |
| Focus group 2 | 64 | Female | White British | Post-graduate degree or equivalent | over 20 years |
|  | 40-49 | Male | White British | Post-graduate degree or equivalent | over 5 years |
|  | 40-49 | Female | Latino/Hispanic | Degree or equivalent | over 2 years |
|  | 66 | Male | White British | Degree or equivalent | 10 years |
|  | 40-49 | Female | White European | Degree or equivalent | over 3 years |
|  | 67 | Female | White British | Post-graduate degree or equivalent | over 20 years |
| **Edgetown** |  |  |  |  |  |
| Focus group 1 | 50-59 | Male | British Asian | Degree or equivalent | 5 years |
|  | 50-59 | Female | British Asian | Degree or equivalent | 20 years |
|  | 50-59 | Female | Indian | Degree or equivalent | 30 years |
|  | 40-49 | Male | Indian | Degree or equivalent | 13 years |
|  | 40-49 | Male | British Asian | Degree or equivalent | 25 years |
|  | 60 | Female | White British | A-Levels or equivalent | 30 years |
| **Edgetown** |  |  |  |  |  |
| Focus group 2 | 50-59 | Female | Indian | GCSEs or equivalent | over 5 years |
|  | 50-59 | Female | Indian | A-Levels or equivalent | over 5 years |
|  | 40-49 | Female | British Indian | Degree or equivalent | over 10 years |
|  | 40-49 | Male | Indian | Degree or equivalent | over 40 years |
|  | 65 | Male | Indian | Degree or equivalent | 12 years |
|  | 50-59 | Female | British Asian | A-Levels or equivalent | 15 years |

**Appendix II: Main questions asked in Phase one topic guide**

**Part One: Exploring the meaning of cognition**

Question 1: We’d like to start by talking about what people understand by ‘cognition’ or ‘brain functioning’. What does that mean to you?

[Brainstorm meaning of cognition]

**Part Two: Perceptions of cognitive change**

Do you notice any changes in your cognition?

**Part Three: Actions that might improve cognition**

To what extent, then, do you think you can take action to influence your cognition/how well your brain is working/other]?

**Ending**

Reflecting on the topics we discussed today, what would you say are the most important issues?

Is there anything else that you would like to add that we did not discuss today and you think it is important to mention?

**Appendix III: Main questions included in Phase two topic guide**

**Part One: Exploring the meaning of cognition**

Question 1: We’d like to start by talking about what people understand by ‘cognition’ or ‘brain functioning’. What does that mean to you?

[Brainstorm meaning of cognition]

**Part Two: Local neighbourhood/environment factors and cognition**

We would like to ask what you think about the relationship between your local environment (local neighbourhood, natural and built environment) and your cognition.

Question 2: What do you think about the influence of your local environment on your cognition, if any?

**Part Three: Evidence about the relationship between cognition and environment/behaviour-related factors**

[Following discussion in part two about people’s perceptions of the factors that influence cognition, the group is informed about factors suggested by research, and the findings in relation to their local area]

[Summary of evidence mentioned:]

Evidence suggests that cognition and brain functioning is influenced, among other things, by different factors in our environment. Evidence suggests there are factors which affect your brain functioning immediately and temporarily, but there is also evidence that some factors may be linked to cognitive problems in the future, so that if you are more exposed to these factors you may be more likely, for example, to experience dementia.

Neighbourhood risk factors linked to cognitive decline/dementia are:

Air pollution

Low social contact

Obesity

Depression

Access to nature and green spaces

Question 3:

What are your thoughts on these factors which might influence peoples’ cognitive health in your local area?

- [Remind of above list of factors or paste into chat as needed]

**Part Four: Actions that might improve cognition**

Question 4: What changes could be made to your local environment that might improve/protect [ cognition]? How might these help?

Question 5: What changes might people make to their own behaviour that could influence their cognition/how well their brain is working?

Question 6: Is there a role for education of the general public about factors that affect cognition? How might the messages best be communicated?

**Ending**

Question 7: Reflecting on the topics we discussed today, what would you say are the most important issues? Or is there anything else that you would like to add?
